# *Pythium insidiosum*: the organism that mimics fungal keratitis

**Published:** 2025-01-31

**Authors:** Bhupesh Bagga, Savitri Sharma, Lakshminarayanan Gowtham

**Affiliations:** 1 Consultant Ophthalmologist: The Ramoji Foundation Centre for Ocular Infection, Shantilal Shanghvi Cornea Institute, LV Prasad Eye Institute, Hyderabad, India.; 2 Director Emeritus: Jhaveri Microbiology Centre, LV Prasad Eye Institute, Hyderabad, India.; 3 Research Assistant Scientist, Chigurupati Nageswara Rao Ocular Pharmacology Research Centre, LV Prasad Eye Institute, Hyderabad, India.

**Microbial keratitis is commonly caused by bacteria and fungi, however, what may appear to be fungal keratitis is sometimes the result of infection with a different microorganism: *Pythium insidiosum*.**

*Pythium insidiosum* belongs to a group of parasitic oomycetes, usually found in aquatic environments. Human infection is rare; however, a lack of awareness of *Pythium* spp. as a cause of microbial keratitis may lead to misdiagnosis as fungal keratitis and inappropriate treatment with antifungals. This has resulted in poor outcomes and the need to resort to therapeutic penetrating keratoplasty or evisceration (Figure 1).^1,2^

**Figure 1:**
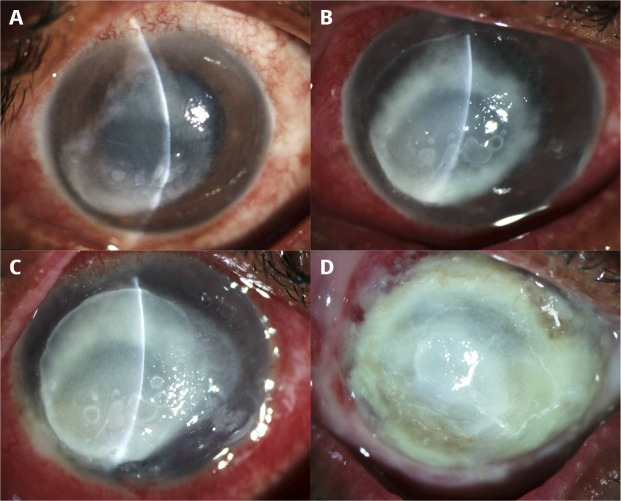
Progression in a patient with *Pythium* keratitis who received antifungals initially. The patient was given a large total penetrating keratoplasty, but this did not eradicate the infection and led to evisceration. **A:** Note the central greyish white ring infiltrate of 7 × 7 mm. **B and C:** Progressive worsening over 10 days. **D:** Total corneal infiltrate with adjacent scleral involvement after one month.

© LV PRASAD CC BY-NC-SA 4.0

*Pythium* spp. produce spores and hyphal structures which led to them being misclassified as moulds until more recent genetic analysis proved otherwise.

The main reason it is important to differentiate *Pythium* infection from fungal infection is because the management is different – *P. insidiosum* is unresponsive to fungal treatment. Instead, it requires antibiotic treatment.

Microbial keratitis that looks like fungal keratitis (both clinically and on microscopy), but which does not respond to antifungal treatment, should be looked at again to rule out *Pythium* keratitis. 

## When to suspect ***Pythium*** keratitis

Although this type of corneal infection is more prevalent in subtropical and tropical climatic regions, the organism is widespread. Males are affected more frequently; this may be because they are more often exposed to ocular trauma during agricultural work. A significant proportion of patients affected have a history of dust or contaminated water in the eye(s), but there have also been reports of patients with a history of contact lens use and exposure to clay particles.

## Signs, symptoms, and clinical findings

Affected patients usually complain of pain, redness, photophobia, and decreased vision on presentation.

The slit lamp findings^3^ include lid oedema and conjunctival congestion, a central or peripheral grayish-white infiltrate with a raised profile (Figure 1), hyphal-like projections, pinhead lesions in the anterior mid-stroma, and peripheral furrowing or guttering around the infiltrate. These features are suggestive of *Pythium* but may be present in some patients with fungal keratitis. Detailed microbiological workup is therefore necessary for diagnosis.

**Figure 2:**
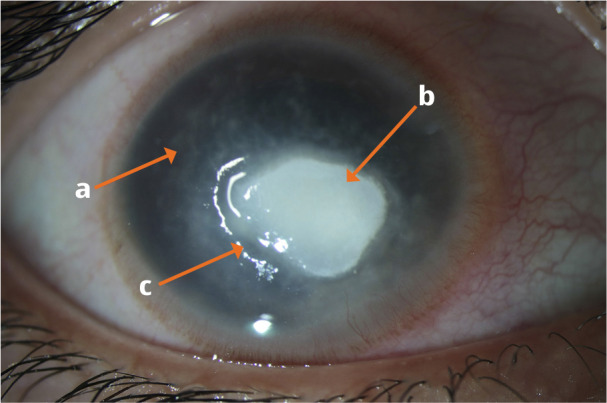
Diffuse slit lamp image of the cornea in a patient recovering from *Pythium* keratitis infection. Hypal-like projections **(a)** and a raised central plaque **(b)** are typical of both fungal and *Pythium* keratitis. As the ulcer starts to heal, there is typically guttering around the infiltrate **(c)**.

There may be associated scleritis and, rarely, endophthalmitis. Intraocular spread is rare, suggesting that the corneal barrier prevents posterior extension of the organism. Peripheral guttering or furrowing around the infiltrate is typically seen as the ulcer heals and the infiltrate size decreases (Figure 2).^4,5^ Significant, deep vascularisation is also seen in this phase.

© LV PRASAD CC BY-NC-SA 4.0

## Diagnosis

The diagnosis of *Pythium* keratitis requires a detailed microbiological workup by an experienced microbiologist.

**Figure 3:**
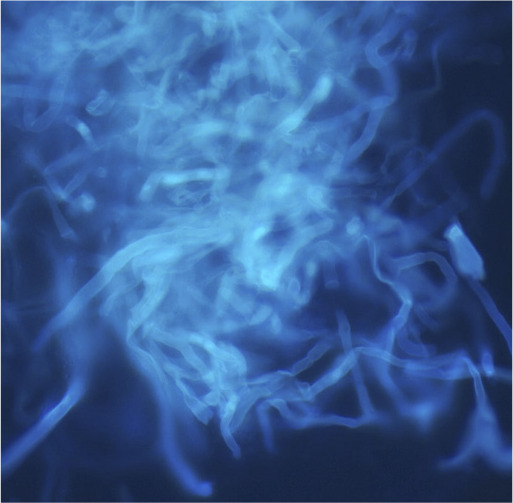
The filaments of *P. insidiosum* are visible on direct microscopy in fluorescent light, on a potassium hydroxide and calcofluor white mount.

*Pythium* filaments can be observed by direct microscopy of a corneal scraping in Gram stain, or in 10% potassium hydroxide (KOH) with 0.1% calcofluor white, viewed under fluorescence (Figure 3). The filaments are classically aseptate or sparsely aseptate,^3^ broad, and ribbon-like. However, this is also characteristic of some filamentous fungi or moulds, so further microbiological workup is needed to make a definitive diagnosis.

© JHAVERI MICROBIOLOGY CENTRE, LV PRASAD EYE INSTITUTE CC BY-NC-SA 4.0

**Figure 4:**
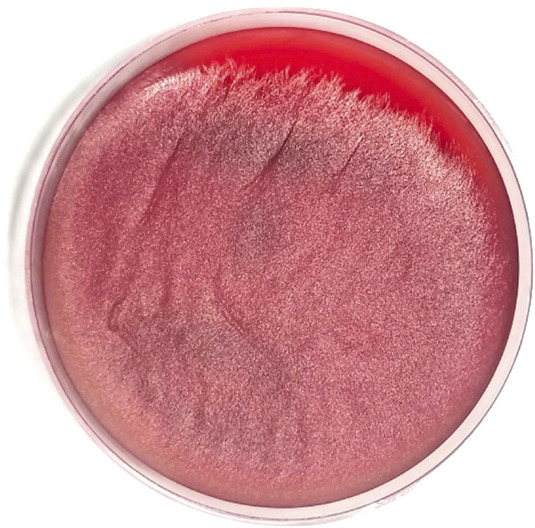
Flat, submerged translucent growth of *P. insidiosum* in blood agar.

*Pythium* keratitis can be differentiated from fungal keratitis (including filamentous fungi or moulds) by staining a corneal scraping smear using a mixture of potassium iodide and iodine with 65% sulphuric acid (IKI-H2SO4). The slide must be examined immediately under the microscope. The *P. insidiosum* filaments are stained bluish-black, whereas fungal filaments appear yellowish.^6^ The growth on blood agar is feathery-edged and flat, and it is colourless or light brown (Figure 4). Further investigation includes growth of zoospores on carnation leaf pieces placed on non-nutrient agar and polymerase chain reaction (PCR).^7^

© JHAVERI MICROBIOLOGY CENTRE, LV PRASAD EYE INSTITUTE CC BY-NC-SA 4.0

## Treatment and management

Treatment is challenging as infection typically takes 2–3 months to resolve and patients may need to be admitted. Non-adherence could become an issue.

Patients presenting in the **early or moderate stages of** *Pythium* **keratitis** should be managed initially with a combination of antibiotics for a minimum of two weeks and observed closely. After the diagnosis is confirmed with a combined clinical and microbiological evaluation, start the patient on a combination of the following:

- Topical linezolid 0.2% (IV preparation) every hour during the day and every two hours at night
- Azithromycin 1% eye ointment twice a day
- Azithromycin 500 mg (orally) once a day for two weeks.

The combined topical medications are continued until a response is noted in the form of scarring or decrease in the size of infiltrate. With timely medical intervention, more than 70% cases can be successfully managed.^3^

Most patients can be seen as outpatients with regular follow-up. Patients who may have difficulty adhering with treatment, those who live far away, and those with only one functioning eye are admitted for 1–2 weeks.

Examine patients clinically every three days, followed by once a week, to assess the response. Check for thinning and deeper extension of infiltrate, including limbal infection, as this suggests the infection might be moving beyond the cornea, and could then spread more widely.

**Figure 5:**
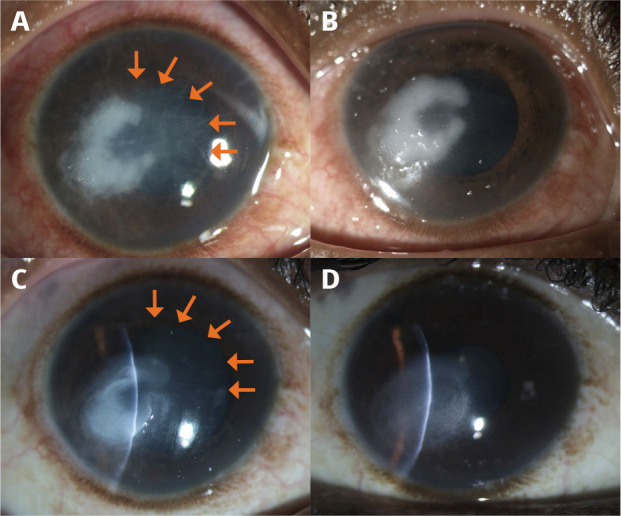
A patient with *Pythium* keratitis. **A:** White mid-stromal infiltrate (5 mm × 6 mm) with tentacular extension (orange arrows) in the surrounding deeper stroma. **B to D:** stages of resolution showing tentacles (orange arrows) disappearing with medical treatment.

**Good response** to medical treatment is evident from the decrease in the size of hyphal-like extensions (Figure 5) and cellularity of the surrounding stroma.

© LV PRASAD EYE INSTITUTE CC BY-NC-SA 4.0

**Poor response** to medical treatment includes:

- **Thinning.** Plan cyanoacrylate glue application in patients with significant corneal thinning to avoid corneal perforation. 
- **Extension of the infiltrate and limbal infection.** Consider therapeutic penetrating keratoplasty.

Patients presenting with **advanced** *Pythium* keratitis need close supervision and should be admitted and seen daily. Therapeutic penetrating keratoplasty with an extra 1.5 mm margin may be needed, as medical treatment alone may not be sufficient for the eradication of infection. Unfortunately, this modality is associated with a high risk of recurrence of infection, and evisceration may eventually be necessary.

The combined topical medications are continued until a response is noted in the form of scarring or decrease in the size of infiltrate. With timely medical intervention, more than 70% of patients can be successfully managed.^3^

## Follow-up care

On the resolution of keratitis, there are increased chances of the development of cataract and glaucoma, which can significantly delay visual recovery, in addition to causing corneal scarring to develop. The development of cataract can be secondary to severe inflammatory reactions due to long-standing infection. Many of these patients need optical keratoplasty and cataract surgery for visual rehabilitation. The outcomes of these grafts are similar to grafts done for other indications.
